# U-shaped relationship of plasma homocysteine with new-onset metabolic dysfunction-associated steatotic liver disease: a cohort study

**DOI:** 10.3389/fnut.2026.1737722

**Published:** 2026-04-29

**Authors:** Yangxuan He, Jingshan Jiang, Jiayi Deng, Fei Xu, Meng Li, Song Leng

**Affiliations:** Health Management Center, Second Hospital of Dalian Medical University, Dalian, China

**Keywords:** cohort study, metabolic dysfunction-associated steatotic liver disease, plasma homocysteine, restricted cubic spline, U-shaped

## Abstract

**Objective:**

Metabolic dysfunction-associated steatotic liver disease (MASLD) is defined by hepatic steatosis accompanied by metabolic abnormalities. Prior cross-sectional studies on the association between plasma homocysteine (Hcy) and MASLD, often limited by small sample sizes, have reported conflicting results.

**Methods:**

This study included 5,184 adults from the Dalian Health Management Cohort (DHMC; 2014–2023), excluding those with baseline MASLD or severe comorbidities. MASLD was diagnosed using liver ultrasonography and metabolic criteria. Hyperhomocysteinemia (HHcy) was defined as a plasma Hcy level >15 μmol/L. Cox proportional hazards models and restricted cubic spline (RCS) analyses evaluated linear and nonlinear associations between baseline Hcy levels and incident MASLD. Subgroup and sensitivity analyses ensured result robustness.

**Results:**

Over a median follow-up of 2.66 years, 1,204 participants developed MASLD. After multivariable adjustment, each standard deviation (SD) increase in Hcy level was associated with a 18% higher risk of incident MASLD (hazard ratio [HR] = 1.18, 95% confidence interval [CI]: 1.11–1.25). Participants with HHcy had a 45% greater risk of developing MASLD compared to those without (HR = 1.45, 95% CI: 1.26–1.68). RCS analysis revealed a U-shaped relationship, with an approximate inflection point around 10 μmol/L identified by restricted cubic spline analysis. Subgroup and sensitivity analyses confirmed these findings.

**Conclusion:**

Plasma Hcy levels demonstrated a U-shaped, independent association with the risk of incident MASLD, suggesting that both very low and high concentrations may adversely impact hepatic metabolic health.

## Introduction

Metabolic dysfunction-associated steatotic liver disease (MASLD) is defined by the presence of hepatic steatosis along with at least one criterion of metabolic dysfunction (1). It affects nearly one-third of the global population, with significant regional variations in prevalence (2, 3). Among Asian countries, China reports the highest incidence and annual mortality of MASLD (4). Current evidence indicates a substantial overlap between nonalcoholic fatty liver disease (NAFLD) and MASLD, as approximately 99% of NAFLD patients can be reclassified as having MASLD (5). This condition has become a major risk factor for liver cirrhosis, hepatocellular carcinoma, type 2 diabetes mellitus (T2DM), chronic kidney disease, and cardiovascular diseases, imposing a growing burden on global health (6–9).

Homocysteine (Hcy), a sulfur-containing amino acid involved in methionine metabolism, is primarily metabolized in the liver (10). Previous cross-sectional and case–control studies have explored the association between Hcy and NAFLD, but the findings remain inconsistent. Some studies have demonstrated a positive correlation between Hcy and MASLD (11–13), while others have suggested sex-specific differences; for instance, a Korean study reported a significant association in men but not in women (14). Conversely, several investigations have observed negative correlations or no significant relationship (15, 16). The predominance of cross-sectional or case–control designs, frequently coupled with limited sample sizes in previous research, has hindered the establishment of a causal relationship between Hcy and MASLD. Therefore, we conducted a large-scale, long-term cohort study to investigate the association between circulating Hcy concentrations and incident MASLD.

## Materials and methods

### Study population

**Figure 1:**
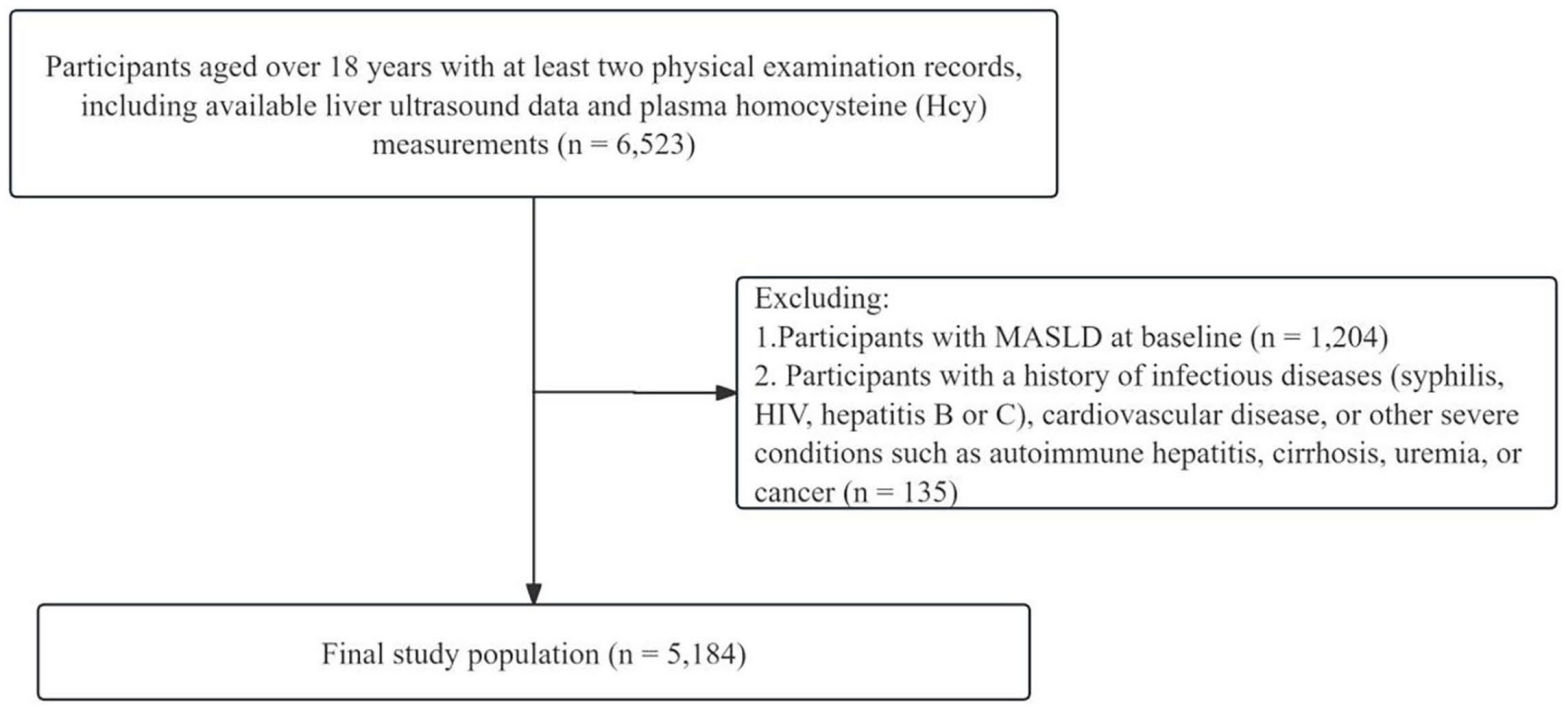
Flowchart of the study population selection. MASLD, Metabolic dysfunction-associated steatotic liver disease; HIV, human immunodeficiency virus.

Participants were drawn from the Dalian Health Management Cohort (DHMC; CCC2023112102), a multicenter cohort initiated in 2014 at the Second Hospital of Dalian Medical University. Adults aged 18 years or older who underwent at least two consecutive annual health examinations between 2014 and 2023, with available liver ultrasound and plasma Hcy data, were eligible for inclusion. The initial cohort comprised 6,523 participants. Exclusion criteria were applied as follows: (1) participants diagnosed with MASLD at baseline (*n* = 1,204); (2) participants with a history of infectious diseases (syphilis, HIV, hepatitis B, or hepatitis C), cardiovascular disease, or severe conditions such as autoimmune hepatitis, cirrhosis, uremia, or cancer (*n* = 135). After exclusions, the final study population consisted of 5,184 participants for the analysis of MASLD incidence (Figure 1). The date of the first health examination was defined as the index date, and participants were followed until either the onset of MASLD or the censoring date of December 31, 2023.

Demographic and clinical information, including sex, age, medical history, smoking status, and alcohol consumption, was collected using standardized questionnaires. Alcohol consumption was assessed using standardized questionnaires. Drinking status (never vs. current/history) was included as a covariate. In addition, alcohol intake was estimated based on self-reported consumption and converted into grams per day, which was used to define excessive alcohol intake according to sex-specific thresholds (≥20 g/day for females and ≥30 g/day for males) in the diagnosis of MASLD. Medication use was defined based on self-reported information. Due to variability in reporting, detailed classification of specific drug classes was not consistently available; therefore, medication use was included as a binary variable (yes/no) in the analyses. Anthropometric measurements were taken with participants in light clothing and without shoes. Waist circumference was measured at the midpoint between the lower rib margin and the iliac crest. Blood pressure was assessed using an Anron electronic sphygmomanometer (HBP9020, Japan) after a five-minute rest. Fasting venous blood samples, collected after at least 8 hours of fasting, were analyzed for biochemical parameters including hemoglobin, platelet count, alanine aminotransferase (ALT), aspartate aminotransferase (AST), serum uric acid, creatinine, fasting plasma glucose (FPG), total cholesterol, triglycerides, high-density lipoprotein cholesterol (HDL-C), low-density lipoprotein cholesterol (LDL-C), and Hcy levels.

Body mass index (BMI) was calculated as weight (kg)/height (m^2^). Smoking and alcohol consumption were categorized as “never” or “current/history of use.” Hypertension was defined as systolic blood pressure ≥140 mmHg and/or diastolic blood pressure ≥90 mmHg, antihypertensive use, or self-reported diagnosis (17). Dyslipidemia was defined as triglycerides >150 mg/dL, total cholesterol >200 mg/dL, LDL-C >130 mg/dL, and/or HDL-C ≤40 mg/dL (18). Hyperhomocysteinemia was defined as plasma Hcy levels >15 μmol/L (10).

### Diagnostic criteria for MASLD

MASLD was diagnosed by qualified ultrasonographers confirming hepatic steatosis via liver ultrasound, excluding other etiologies or excessive alcohol consumption (≥20 g/day for females or ≥30 g/day for males). Participants were required to exhibit at least one of the following cardiometabolic risk factors: (1) BMI ≥23 kg/m^2^ or waist circumference ≥90 cm (males) or ≥80 cm (females); (2) FPG ≥5.6 mmol/L, glycated hemoglobin ≥5.7%, or a history of type 2 diabetes mellitus (T2DM) or ongoing T2DM treatment; (3) blood pressure ≥130/85 mmHg or receiving antihypertensive medications; (4) TG ≥1.70 mmol/L or receiving lipid-lowering therapy; (5) HDL-C <1.0 mmol/L (males) or <1.3 mmol/L (females) or receiving lipid-lowering treatment (5, 9).

### Sample size

Based on the “10 events per variable” principle, this study included 11 covariates, requiring a minimum of 110 outcome events. With 1,204 incident MASLD cases observed, the study had sufficient statistical power to support multivariable regression analyses and reduce the risk of model overfitting (19).

### Ethical statement

The study adhered to the Declaration of Helsinki, was approved by the Institutional Ethics Committee (Approval No. KY2025-603-01), and waived informed consent due to its retrospective, anonymized nature (20, 21). Patient data were protected through encryption and strict privacy protocols.

### Methods and statistical analysis

Normally distributed variables are reported as mean ± standard deviation, while non-normally distributed variables are presented as median (25th–75th percentiles). Standardized mean differences (SMDs) were additionally calculated to evaluate the magnitude of baseline differences between groups, as SMDs are less sensitive to sample size than traditional significance testing. SMDs are presented as absolute values, with a value >0.1 indicating meaningful imbalance. Extreme values were handled using winsorization based on the interquartile range (IQR) method (lower bound: Q1 − 1.5 × IQR; upper bound: Q3 + 1.5 × IQR) to reduce the influence of outliers. Missing covariate data were imputed using a random forest-based approach under the assumption of missing at random (MAR) (22). The proportions of missing data for key covariates are provided in Supplementary Table 1.

Kaplan–Meier curves were generated to estimate the cumulative incidence of MASLD across homocysteine categories (<10, 10–15, and >15 μmol/L), and differences between groups were evaluated using the log-rank test. Cox proportional hazards regression was used to assess the association between baseline Hcy levels and MASLD risk. Model 1 was unadjusted, Model 2 was adjusted for age and sex, and Model 3 was further adjusted for age, sex, smoking, drinking, drug use, T2DM, hypertension, dyslipidemia, uric acid, BMI, and eGFR. Covariates were selected based on prior literature, clinical relevance, and their potential roles as confounders in the association between Hcy and MASLD. Restricted cubic spline (RCS) analysis was applied to explore the potential nonlinear association between Hcy and MASLD risk, using four knots placed at the 5th, 35th, 65th, and 95th percentiles. Nonlinearity was assessed using a likelihood ratio test comparing the model with only the linear term to the model including spline terms. To further characterize the dose–response relationship, a two-piecewise linear regression model was fitted to estimate potential threshold effects. Subgroup analyses were conducted according to age, sex, BMI, T2DM, hypertension, and dyslipidemia status. Sensitivity analyses were performed by excluding participants with less than 2 years of follow-up or early MASLD onset, as well as participants with medication use, to reduce reverse causation and pharmacologic confounding. All analyses were performed using Stata version 18 and R version 4.3.2, with a two-sided *p*-value of less than 0.05 considered statistically significant.

## Results

### Baseline characteristics

During a median follow-up of 2.66 years, 1,204 out of 5,184 participants developed MASLD. Among them, 728 were in the hyperhomocysteinemia (HHcy) group and 4,456 in the non-HHcy group. Absolute SMDs were used to evaluate baseline differences between groups, with an SMD of 0.1 considered the threshold for meaningful imbalance (Table 1). Variables with absolute SMDs >0.1 included age, sex, SBP, DBP, height, WC, weight, BMI, medication use, type 2 diabetes, dyslipidemia, hypertension, neutrophils, lymphocytes, monocytes, WBC, RBC, hemoglobin, ALT, AST, uric acid, creatinine, eGFR, glucose, triglycerides, HDL-C, LDL-C, and MASLD. In contrast, smoking, alcohol consumption, platelet count, and total cholesterol showed relatively small between-group differences, with absolute SMDs ≤0.1. Baseline comparisons between the MASLD and non-MASLD groups are presented in Supplementary Table 2.

**Table 1:** Baseline characteristics of the study population according to hyperhomocysteinemia status.

Characteristics	Hyperhomocysteinemia status			Absolute SMD (95% CI)
	Overall (*n* = 5,184)	No hyperhomocysteinemia (*n* = 4,456)	Hyperhomocysteinemia (*n* = 728)	
Age, years	41.00 (34.00, 51.00)	41.00 (34.00, 51.00)	41.00 (33.00, 53.00)	0.13 (0.20, 0.07)
Sex, *n* (%)				0.48 (0.42, 0.55)
Male	2,451 (47)	1,829 (41)	622 (85)	
Female	2,733 (53)	2,627 (59)	106 (15)	
SBP, mmHg	122.00 (111.00, 132.00)	121.00 (111.00, 131.00)	125.00 (115.00, 135.00)	0.37 (0.43, 0.30)
DBP, mmHg	74.00 (67.00, 81.00)	74.00 (67.00, 81.00)	76.00 (70.00, 84.00)	0.36 (0.42, 0.29)
Height, cm	168.00 (162.00, 175.00)	167.00 (162.00, 173.00)	174.00 (168.50, 178.00)	0.33 (0.39, 0.26)
WC, cm	81.00 (74.00, 88.00)	80.00 (73.00, 87.00)	86.00 (80.00, 92.00)	0.89 (0.96, 0.82)
Weight, kg	65.00 (57.00, 74.00)	64.00 (56.00, 73.00)	72.00 (65.00, 79.00)	0.87 (0.93, 0.80)
BMI, kg/m^2^	22.99 (21.07, 25.14)	22.83 (20.96, 24.98)	23.92 (22.07, 25.98)	0.90 (0.96, 0.83)
Smoking, *n* (%)	814 (16)	671 (15)	143 (20)	0.07 (0.00, 0.13)
Alcohol consumption, *n* (%)	752 (15)	633 (14)	119 (16)	0.02 (0.04, 0.09)
Medication use, *n* (%)	393 (7.6)	314 (7.0)	79 (11)	0.23 (0.17, 0.30)
Type 2 diabetes, *n* (%)	225 (4.3)	183 (4.1)	42 (5.8)	0.13 (0.06, 0.19)
Dyslipidemia, *n* (%)	1,168 (23)	925 (21)	243 (33)	0.45 (0.38, 0.51)
Hypertension, *n* (%)	791 (15)	637 (14)	154 (21)	0.24 (0.17, 0.30)
Neutrophils, ×10^9^/L	3.26 (2.66, 4.00)	3.22 (2.63, 3.95)	3.45 (2.86, 4.33)	0.29 (0.35, 0.22)
Lymphocytes, ×10^9^/L	1.86 (1.56, 2.22)	1.86 (1.55, 2.21)	1.91 (1.59, 2.30)	0.26 (0.33, 0.20)
Monocytes, ×10^9^/L	0.32 (0.25, 0.40)	0.31 (0.24, 0.39)	0.35 (0.28, 0.44)	0.20 (0.26, 0.13)
WBC, ×10^9^/L	5.64 (4.87, 6.62)	5.60 (4.81, 6.55)	5.90 (5.18, 6.99)	0.35 (0.42, 0.29)
RBC, ×10^12^/L	4.73 (4.43, 5.07)	4.69 (4.41, 5.01)	5.03 (4.70, 5.28)	0.47 (0.53, 0.40)
Hemoglobin, g/L	143.00 (133.00, 155.00)	141.00 (132.00, 153.00)	154.00 (145.00, 161.00)	0.45 (0.52, 0.39)
Platelets, ×10^9^/L	237.00 (205.00, 274.00)	237.00 (206.00, 275.00)	233.00 (202.00, 269.00)	0.06 (0.12, 0.01)
ALT, U/L	16.90 (12.91, 23.16)	16.56 (12.61, 22.55)	18.86 (14.42, 26.00)	0.47 (0.54, 0.41)
AST, U/L	19.15 (16.46, 22.73)	19.08 (16.34, 22.63)	19.64 (16.99, 23.61)	0.16 (0.23, 0.10)
Uric acid, μmol/L	331.00 (273.94, 398.19)	320.31 (268.33, 384.36)	394.09 (336.40, 456.40)	0.58 (0.65, 0.52)
Creatinine, μmol/L	65.73 (56.09, 78.02)	63.28 (55.17, 75.32)	78.96 (70.80, 87.62)	0.33 (0.39, 0.26)
eGFR, mL/min/1.73 m^2^	109.85 (100.67, 117.64)	110.33 (101.02, 118.29)	108.28 (99.44, 115.30)	0.16 (0.10, 0.22)
Glucose, mmol/L	5.40 (5.13, 5.73)	5.38 (5.12, 5.71)	5.50 (5.18, 5.82)	0.34 (0.41, 0.28)
Total cholesterol, mmol/L	4.88 (4.31, 5.52)	4.88 (4.30, 5.52)	4.91 (4.35, 5.54)	0.08 (0.14, 0.02)
Triglycerides, mmol/L	1.27 (0.93, 1.72)	1.24 (0.91, 1.69)	1.46 (1.07, 1.91)	0.64 (0.70, 0.57)
HDL-C, mmol/L	1.39 (1.18, 1.63)	1.42 (1.21, 1.65)	1.24 (1.07, 1.45)	0.68 (0.62, 0.75)
LDL-C, mmol/L	2.67 (2.20, 3.19)	2.64 (2.18, 3.17)	2.83 (2.32, 3.35)	0.19 (0.25, 0.12)
MASLD, *n* (%)	1,204 (23)	938 (21)	266 (37)	0.35 (0.27, 0.43

SBP, systolic blood pressure; DBP, diastolic blood pressure; WC, waist circumference; WBC, white blood cell count; RBC, red blood cell count; ALT, alanine aminotransferase; AST, aspartate aminotransferase; eGFR, estimated glomerular filtration rate; HDL-C, high-density lipoprotein cholesterol; LDL-C, low-density lipoprotein cholesterol; MASLD, metabolic dysfunction-associated steatotic liver disease.

### Kaplan–Meier curves and cox regression results

**Figure 2:**
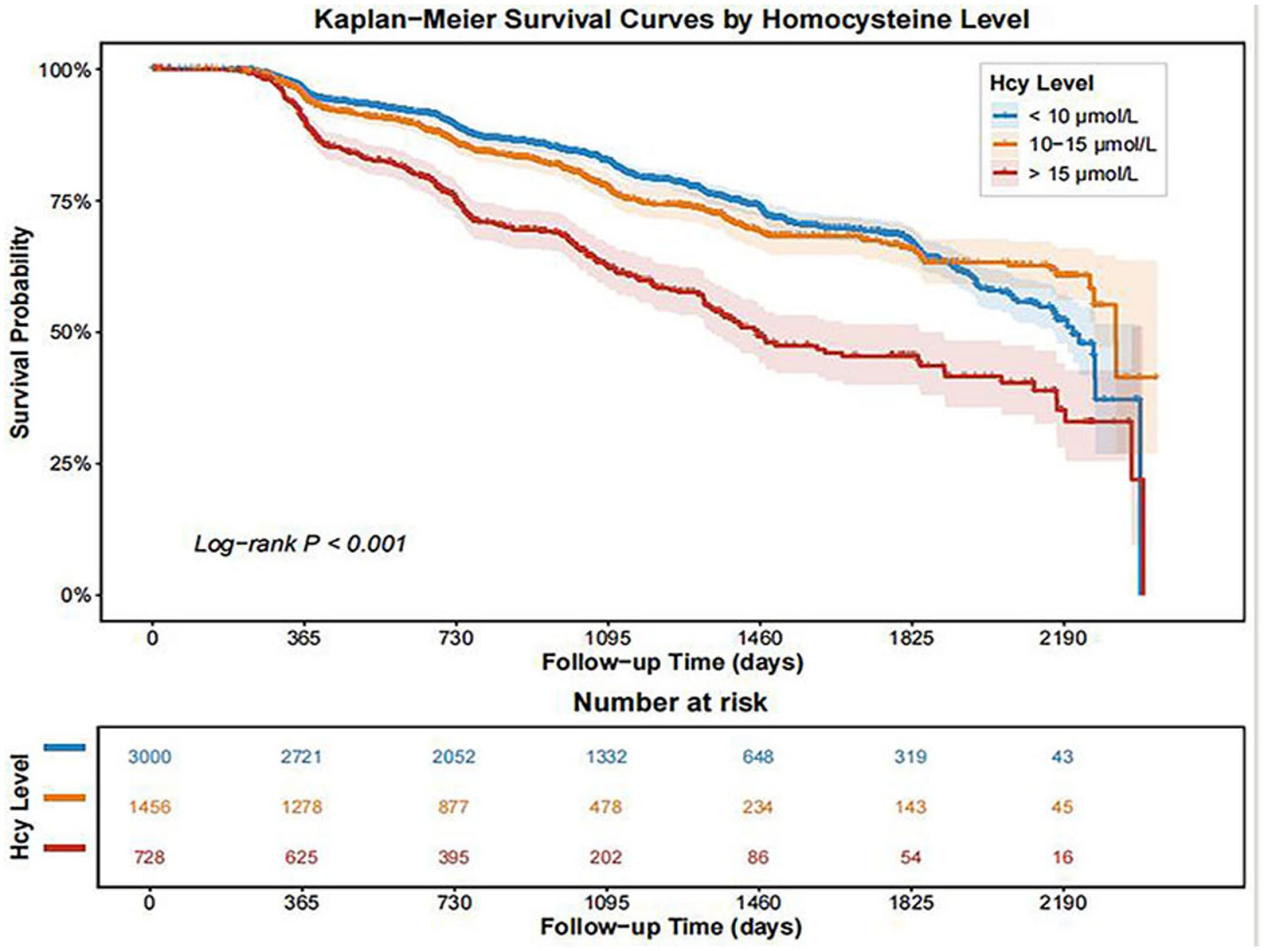
Kaplan–Meier survival curves for incident MASLD according to baseline homocysteine categories. MASLD, Metabolic dysfunction-associated steatotic liver disease.

Kaplan–Meier analysis demonstrated significant differences in the cumulative incidence of MASLD across homocysteine categories (log-rank *p* < 0.001, Figure 2). A graded relationship was observed, with progressively higher MASLD incidence as Hcy levels increased. Participants in the >15 μmol/L group exhibited the highest risk, followed by those in the 10–15 μmol/L group, while the <10 μmol/L group had the lowest incidence. A variance inflation factor (VIF) analysis was conducted to assess potential multicollinearity among covariates, and no significant multicollinearity was detected (Supplementary Table 3). Cox regression analysis further confirmed a positive association between Hcy and MASLD risk (Table 2). The proportional hazards assumption was satisfied, supporting the validity of the Cox model. For each 1-SD increase in Hcy, the risk of MASLD increased significantly across all models (Model 3: HR = 1.18, 95% CI: 1.11–1.25, *p* < 0.001). Similarly, participants with HHcy had a higher risk of MASLD compared to those without. This association remained significant after full adjustment (Model 3: HR = 1.45, 95% CI: 1.26–1.68, *p* < 0.001).

**Table 2:** Cox regression analysis for homocysteine and MASLD risk.

Variable	Model 1		Model 2		Model 3	
	HR (95% CI)	*p*-value	HR (95% CI)	*p*-value	HR (95% CI)	*p-*value
Per SD increment	1.41 (1.34–1.48)	<0.001	1.20 (1.13–1.28)	<0.001	1.18 (1.11–1.25)	<0.001
Hyperhomocysteinemia	2.07 (1.81–2.37)	<0.001	1.52 (1.3–1.75)	<0.001	1.45 (1.26–1.68)	<0.001

Model 1: Unadjusted; Model 2: Adjusted for age and sex; Model 3: Adjusted for age, sex, smoking, drinking, drug use, T2DM, hypertension, dyslipidemia, uric acid, BMI, and eGFR.

HR, hazard ratio; CI, confidence interval.

### Restricted cubic spline and threshold effect analysis

**Figure 3:**
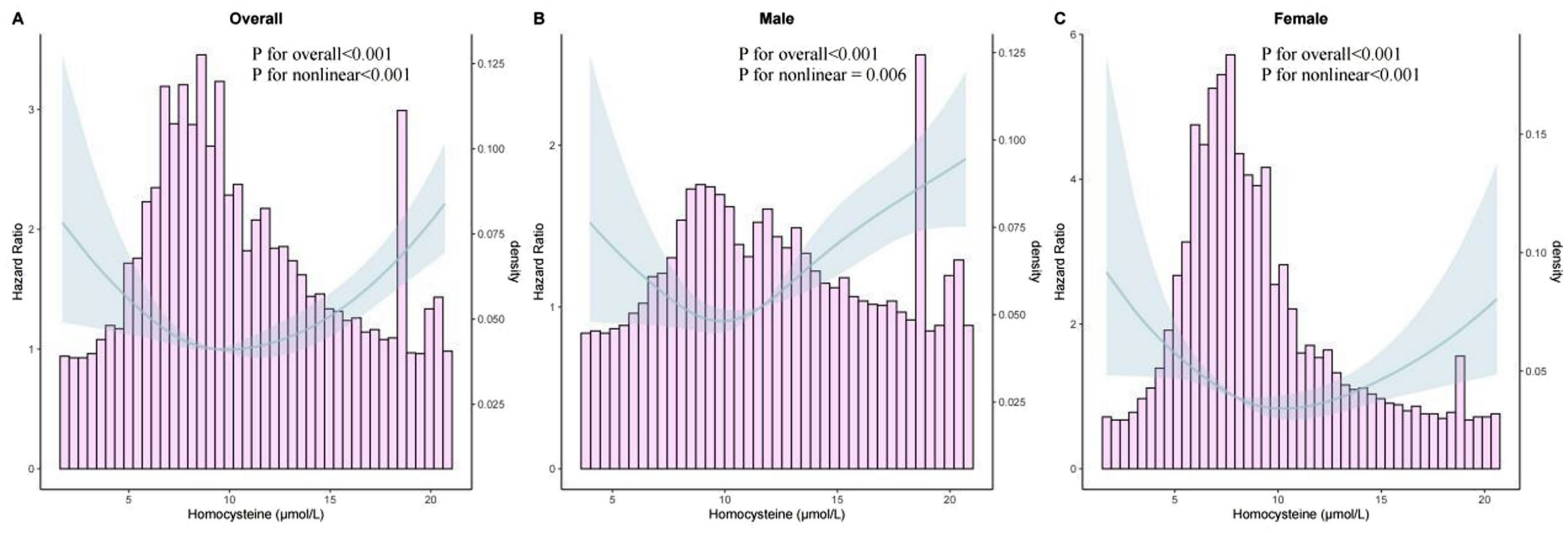
The dose–response relationship between homocysteine levels and the risk of MASLD. **(A)** Overall population; **(B)** Male subgroup; **(C)** Female subgroup.

RCS analysis demonstrated a U-shaped relationship between Hcy levels and MASLD incidence in the overall population as well as in both male and female subgroups (Figure 3), with significant nonlinearity (*p* < 0.05). Threshold effect analysis further supported a nonlinear relationship between Hcy and MASLD risk (Table 3). Using a two-piecewise linear regression model, an inflection point was identified at approximately 10 μmol/L. In the linear model, Hcy was positively associated with MASLD risk (HR = 1.036, 95% *CI*: 1.020–1.051, *p* < 0.001). Segmented linear modeling revealed that when Hcy levels were below 10 μmol/L, a negative association was observed (HR = 0.896, 95% CI: 0.855–0.938, *p* < 0.001); when levels exceeded 10 μmol/L, a significant positive association emerged (HR = 1.082, 95% CI: 1.060–1.103, *p* < 0.001). The likelihood ratio test (*p* < 0.001) confirmed this nonlinear pattern. Details are provided in Table 3.

**Table 3:** Threshold effect analysis of homocysteine on MASLD risk using a two-piecewise linear regression model.

Variables	Effect size, 95% CI	*p-*value
Model 1: single linear Cox model	1.036 (1.020–1.051)	<0.001
Model 2: two-piecewise Cox model		
Inflection point	10	
<10 (μmol/L)	0.896 (0.855–0.938)	<0.001
≥10 (μmol/L)	1.082 (1.060–1.103)	<0.001
*p*-for likelihood ratio test		<0.001

Model 1 represents the single linear Cox model; Model 2 represents the two-piecewise Cox model with an inflection point at 10 μmol/L. Effect sizes are presented as hazard ratios with 95% confidence intervals (CI).

Hcy, homocysteine; CI, confidence interval.

### Baseline biochemical characteristics according to homocysteine categories

As shown in Supplementary Table 4, participants with higher homocysteine levels exhibited a less favorable metabolic profile. Compared with the Hcy < 10 μmol/L group, those with Hcy > 15 μmol/L had higher median levels of BMI (23.92 vs. 22.57 kg/m^2^), triglycerides (1.46 vs. 1.20 mmol/L), LDL-C (2.83 vs. 2.61 mmol/L), ALT (18.86 vs. 16.03 U/L), glucose (5.50 vs. 5.35 mmol/L), uric acid (394.09 vs. 302.05 μmol/L), and creatinine (78.96 vs. 59.52 μmol/L), while HDL-C (1.24 vs. 1.45 mmol/L) and eGFR (104.49 vs. 112.74 mL/min/1.73 m^2^) were lower.

The standardized mean differences suggested that the most pronounced differences across homocysteine categories were observed for creatinine (SMD for <10 vs. >15 = 1.367), uric acid (1.013), and eGFR (0.731), followed by HDL-C (0.659). These findings indicate that elevated homocysteine levels were associated with concurrent disturbances in lipid metabolism, liver enzymes, and renal function.

### Subgroup analysis

**Figure 4:**
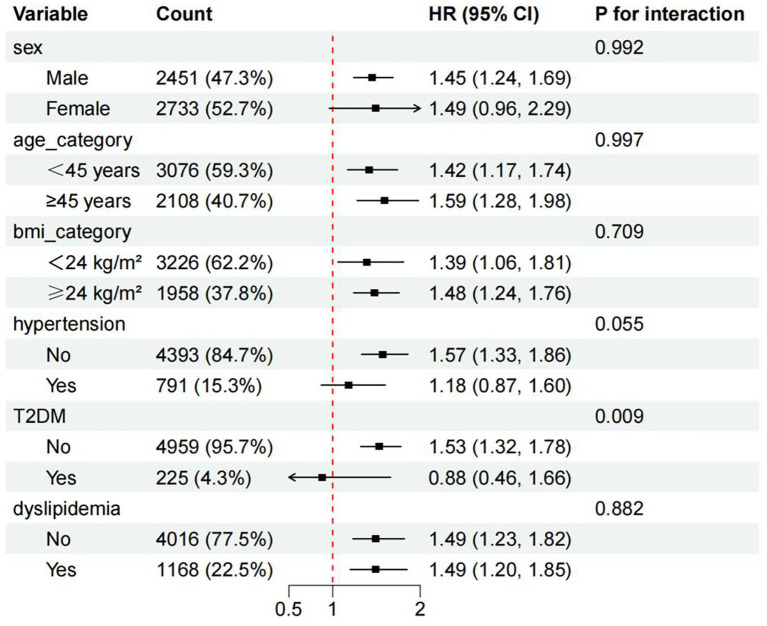
Subgroup analysis of MASLD risk. BMI, Body mass index; T2DM, type 2 diabetes mellitus; MASLD, metabolic dysfunction-associated steatotic liver disease.

Subgroup analyses indicated that HHcy was positively associated with MASLD incidence across most subgroups, with attenuated associations observed among females and individuals with hypertension. A statistically significant interaction was identified for type 2 diabetes mellitus (*p*-for interaction = 0.009), while no significant interaction was observed for hypertension (*p*-for interaction = 0.055). Detailed results are presented in Figure 4.

### Sensitivity analysis

Sensitivity analyses were performed after excluding 393 participants using medications and 632 participants who developed MASLD within the first 2 years. The results remained consistent with the main findings. Detailed results are shown in Supplementary Table 5.

## Discussion

Based on data from the large-scale Dalian Health Management Cohort, our study demonstrated that the incidence of MASLD was significantly higher among individuals with HHcy compared with those without. Cox regression analysis revealed a robust positive association between baseline Hcy levels and MASLD risk, which persisted after comprehensive multivariable adjustment. Restricted cubic spline (RCS) analysis further uncovered a U-shaped relationship between Hcy and MASLD incidence, with an inflection point around 10 μmol/L. Notably, this value reflects a data-driven inflection point rather than a clinical diagnostic threshold. Both low and high Hcy levels were associated with an increased risk of MASLD. Subgroup analyses indicated that this relationship remained consistent across most populations, although it appeared weaker among females and individuals with hypertension. A significant effect modification by type 2 diabetes mellitus was observed. Sensitivity analyses yielded similar results, underscoring the stability and reliability of the findings. In addition, model assumptions were formally tested, and multiple analytical approaches were applied to ensure the robustness and consistency of the results.

Some previous cross-sectional studies have found a positive correlation between Hcy levels and MASLD prevalence (12, 23–26). Early observational studies showed that patients with MASLD had higher Hcy levels than healthy controls, and that elevated Hcy was independently associated with increased disease risk (12, 25, 27). More recent observational and meta-analytic evidence has further reinforced this pattern, indicating that higher Hcy levels are associated with greater odds of MASLD and liver fibrosis (23, 28). In addition, hyperhomocysteinemia has been directly linked to MASLD, and Hcy-based multimarker models have shown improved predictive performance for MASLD detection (13, 29). Nevertheless, the evidence remains inconsistent. Chen et al. reported no significant causal association between genetically predicted plasma homocysteine levels and NAFLD, NASH, or cirrhosis in a Mendelian randomization analysis (30). Conversely, small-sample studies by Polyzos et al. (15) and Xu et al. (16) observed reduced Hcy levels in MASLD patients compared with healthy controls, leading to seemingly contradictory conclusions. In addition, some cohort studies have suggested significant sex-specific differences in this association. For instance, Won et al. (14) reported a significant relationship between Hcy and MASLD risk in men but not in women in a Korean population, suggesting that sex-specific metabolic or hormonal factors might modulate the role of Hcy in hepatic lipid metabolism. The U-shaped relationship identified in our cohort provides a plausible explanation for these discrepancies, suggesting that both hyperhomocysteinemia and excessively low Hcy levels may be detrimental to liver health. This non-linear pattern aligns with observations in other metabolic disorders. For example, Wang et al. (31) reported a U-shaped association between Hcy and incident hypertension, implying that both Hcy deficiency and excess may contribute to pathophysiological imbalances. In the present study, individuals with higher homocysteine levels showed a consistently less favorable biochemical profile, including higher BMI, triglycerides, LDL-C, ALT, glucose, uric acid, and creatinine, together with lower HDL-C and eGFR. These findings suggest that elevated homocysteine may cluster with multiple metabolic abnormalities relevant to MASLD. In particular, the inverse association with HDL-C and the positive associations with triglycerides and liver enzymes support the notion that hyperhomocysteinemia may reflect a metabolically adverse state characterized by dyslipidemia and hepatic injury. In addition, the marked differences in creatinine, uric acid, and eGFR imply a close relationship between homocysteine and renal function, which should be considered when interpreting the association between homocysteine and MASLD.

Homocysteine, a key intermediate in methionine metabolism, exerts multifaceted effects on hepatic lipid homeostasis. Elevated Hcy concentrations may interfere with the methylation of phosphatidylcholine (PC) and sterol regulatory element-binding proteins (SREBPs) (26). PC plays a pivotal role in maintaining lipid transport and membrane integrity, and reduced PC levels can disrupt very-low-density lipoprotein (VLDL) secretion, leading to hepatic lipid accumulation (32). Furthermore, SREBP-1a, a transcription factor essential for phospholipid synthesis, is activated under conditions of low PC availability, thereby upregulating lipogenic gene expression and promoting lipid droplet formation (33, 34). This process initiates hepatocellular fat deposition, an early event in MASLD pathogenesis. Elevated Hcy also triggers endoplasmic reticulum (ER) stress and activates the unfolded protein response (UPR). When the UPR fails to restore homeostasis, it induces inflammation, apoptosis, and lipid accumulation, creating a self-perpetuating cycle of ER dysfunction and hepatocellular injury (35). Additionally, high Hcy levels can reduce intracellular glutathione synthesis, compromising antioxidant defenses and rendering hepatocytes more vulnerable to reactive oxygen species (ROS) (11). Oxidative stress, characterized by an imbalance between pro-oxidants and antioxidants, leads to mitochondrial dysfunction, lipid peroxidation, and DNA damage (36). These alterations amplify hepatic inflammation, stellate cell activation, and fibrosis, thereby accelerating MASLD progression. Recent mechanistic studies further support the biological plausibility of these observations. One-carbon metabolism appears to be broadly disturbed in MASLD, and these abnormalities may be modifiable by semaglutide treatment (37). Experimental work has also shown that betaine can alleviate NAFLD through BHMT/FTO/m6A/PGC1α-related pathways, linking Hcy remethylation to hepatic lipid handling, whereas additional metabolic studies suggest that sulfur-amino-acid and methionine-cycle regulation may influence steatosis, oxidative stress, and fibrogenesis. These data strengthen the view that Hcy is not merely a passive marker, but part of a broader one-carbon metabolic network relevant to MASLD pathophysiology.

Interestingly, both excessive and deficient Hcy states appear metabolically unfavorable. Studies have shown that both methionine supplementation and deficiency can induce molecular abnormalities associated with hepatic steatosis, including disruptions in one-carbon metabolism and increased oxidative and ER stress (36, 38). Experimental evidence further supports that high Hcy concentrations promote oxidative stress, fibrosis, and inflammatory infiltration (39), whereas abnormally low Hcy levels may reflect impaired methylation potential, leading to reduced phosphatidylcholine synthesis and compromised antioxidant capacity, thereby promoting hepatic lipid accumulation and oxidative stress (40). In addition, low Hcy levels may not necessarily indicate a metabolically favorable state but rather reflect underlying metabolic disturbances (41). Reduced Hcy may be associated with impaired remethylation capacity and decreased availability of methionine and S-adenosylmethionine, which are essential for hepatic lipid export and cellular methylation processes (36). Moreover, given that homocysteine metabolism is closely linked to nutritional status and muscle metabolism, lower Hcy levels may also be observed in conditions such as methionine deficiency, inadequate B-vitamin status, weight loss, sarcopenia, or subclinical malnutrition (42). These states may be accompanied by reduced hepatic metabolic reserve and impaired antioxidant defense, which may increase susceptibility to hepatic steatosis. Collectively, these findings suggest that maintaining Hcy homeostasis is essential for optimal hepatic metabolic function and may explain the U-shaped curve observed in our study.

Our results indicate that plasma Hcy may serve as a potential biomarker for MASLD risk prediction. The threshold effect analysis identified an optimal Hcy level of approximately 10 μmol/L associated with the lowest risk of MASLD. Maintaining Hcy within this range may be relevant for hepatic metabolic stability. Evidence from animal models suggests that dietary modulation of Hcy may influence steatotic changes, indicating potential biological relevance (43, 44). Lifestyle and nutritional factors, such as folate and vitamins B6 and B12, alcohol intake, and body weight, are known to influence homocysteine metabolism and may be associated with MASLD risk. However, these factors were not comprehensively assessed in the present study; therefore, their roles should be interpreted as hypothetical, and residual confounding cannot be excluded.

The present study possesses several strengths. It utilized a large sample size and long-term follow-up, providing substantial statistical power and temporal clarity for causal inference. The cohort design enabled the identification of a novel non-linear association between plasma Hcy and MASLD incidence. However, several limitations merit consideration. Firstly, MASLD diagnosis relied on liver ultrasonography rather than biopsy, which is operator-dependent and has limited sensitivity for detecting mild steatosis, potentially leading to underestimation of MASLD incidence. Secondly, dynamic changes in Hcy, folate, and B-vitamin status were not monitored, and alcohol intake was not comprehensively assessed, limiting mechanistic interpretation and potentially introducing residual confounding. Thirdly, medication use was treated as a single category due to limited detail on specific drug classes, which may have reduced the clinical interpretability of the findings. Fourthly, Hcy levels were assessed only at baseline, and changes over time were not captured, which may have introduced measurement bias. Fifthly, the relatively short follow-up duration may have limited the ability to fully capture long-term MASLD incidence. Sixthly, the study population consisted primarily of Northern Chinese adults, potentially restricting generalizability to other ethnic or geographic populations. Finally, as an observational study, residual confounding cannot be entirely excluded despite rigorous adjustment.

## Conclusion

In conclusion, this study provides the first large-scale evidence that elevated plasma homocysteine levels are a significant risk factor for metabolic dysfunction-associated steatotic liver disease, and that the relationship between Hcy and MASLD follows a U-shaped pattern. Both low and high Hcy concentrations are associated with increased disease risk, suggesting that maintaining moderate Hcy levels may protect hepatic metabolic health. Future multicenter and mechanistic studies are warranted to elucidate the causal pathways linking Hcy to MASLD onset and progression and to evaluate the potential of Hcy-targeted interventions in preventing or mitigating metabolic liver disease.

## Data Availability

The raw data supporting the conclusions of this article will be made available by the authors, without undue reservation.

## Glossary

MASLD: metabolic dysfunction-associated steatotic liver disease
Hcy: homocysteine
HHcy: hyperhomocysteinemia
DHMC: Dalian Health Management Cohort
NAFLD: nonalcoholic fatty liver disease
T2DM: type 2 diabetes mellitus
Hb: hemoglobin
ALT: alanine aminotransferase
AST: aspartate aminotransferase
SUA: serum uric acid
Scr: serum creatinine
FPG: fasting plasma glucose
TC: total cholesterol
TG: triglycerides
HDL-C: high-density lipoprotein cholesterol
LDL-C: low-density lipoprotein cholesterol
BMI: body mass index
SBP: systolic blood pressure
DBP: diastolic blood pressure
IQR: interquartile range
RCS: restricted cubic spline
HR: hazard ratio
CI: confidence interval
PC: phosphatidylcholine
SREBP: sterol regulatory element-binding protein
UPR: unfolded protein response
ROS: reactive oxygen species
RNS: reactive nitrogen species
RBC: red blood cells
