# Supplementary material for: U-shaped relationship of plasma homocysteine with new-onset metabolic dysfunction-associated steatotic liver disease: a cohort study
Source: Front Nutr. 2026 Apr 29;13:1737722. doi: 10.3389/fnut.2026.1737722 (PMC13168133; doi:10.3389/fnut.2026.1737722)
Supplement: Supplementary file 1 [file Data_Sheet_1.docx]

**Supplementary Tables**

**Supplementary Table 1. Summary of Missing Data Across Study Variables**

**Supplementary Table 2. The baseline characteristics based on follow-up outcomes with or without MASLD**

**Supplementary Table 3. Ulticollinearity Test using Variance Inflation Factor**

**Supplementary Table 4. Baseline biochemical characteristics according to homocysteine categories**

**Supplementary Table 5. Sensitivity Analyses for Homocysteine and MASLD Risk**

**Supplementary Table 1. Summary of Missing Data Across Study Variables**

| **Study variable** | **No. of missing values** | **Missing proportion (%)** |
| --- | --- | --- |
| Drinking status | 649 | 12.52 |
| Smoking status | 632 | 12.19 |
| Drug exposure | 619 | 11.94 |
| Waist circumference | 300 | 5.79 |
| Aspartate aminotransferase | 292 | 5.63 |
| Body weight | 253 | 4.88 |
| Height | 252 | 4.86 |
| Body mass index | 178 | 3.43 |
| Diastolic blood pressure | 105 | 2.03 |
| Systolic blood pressure | 104 | 2.01 |
| High-density lipoprotein cholesterol | 58 | 1.12 |
| Serum creatinine | 42 | 0.81 |
| Alanine aminotransferase | 24 | 0.46 |
| Absolute neutrophil count | 22 | 0.42 |
| Absolute lymphocyte count | 22 | 0.42 |
| Absolute monocyte count | 22 | 0.42 |
| White blood cell count | 22 | 0.42 |
| Red blood cell count | 22 | 0.42 |
| Hemoglobin | 22 | 0.42 |
| Platelet count | 22 | 0.42 |
| Blood glucose | 22 | 0.42 |
| Total cholesterol | 16 | 0.31 |
| Triglycerides | 16 | 0.31 |
| Low-density lipoprotein cholesterol | 16 | 0.31 |
| Uric acid | 12 | 0.23 |
| Sex | 0 | 0.00 |
| Type 2 diabetes mellitus | 0 | 0.00 |
| Dyslipidemia | 0 | 0.00 |
| Hypertension | 0 | 0.00 |
| Age | 0 | 0.00 |
| Homocysteine | 0 | 0.00 |

This table summarizes the number and percentage of missing values for each study variable included in the analytic dataset.

**Abbreviations:** ALT, alanine aminotransferase; AST, aspartate aminotransferase; BMI, body mass index; DBP, diastolic blood pressure; HDL, high-density lipoprotein; LDL, low-density lipoprotein; SBP, systolic blood pressure

**Supplementary Table 2. The baseline characteristics based on follow-up outcomes with or without MASLD**

| Characteristics | MASLD Status | | | Absolute SMD (95% CI) |
| --- | --- | --- | --- | --- |
|  | Overall  (n=5,184) | No MASLD  (n=3,980) | MASLD  (n=1,204) |  |
| Age, years | 41.00 (34.00, 51.00) | 41.00 (33.00, 51.00) | 44.00 (36.00, 52.00) | **0.13 (0.07, 0.20)** |
| Sex, n (%) |  |  |  | **0.48 (0.42, 0.55)** |
| Male | 2,451 (47) | 1,665 (42) | 786 (65) |  |
| Female | 2,733 (53) | 2,315 (58) | 418 (35) |  |
| SBP, mmHg | 122.00 (111.00, 132.00) | 120.00 (110.00, 131.00) | 126.00 (116.00, 136.00) | **0.37 (0.30, 0.43)** |
| DBP, mmHg | 74.00 (67.00, 81.00) | 73.00 (66.00, 80.00) | 77.00 (71.00, 84.00) | **0.36 (0.29, 0.42)** |
| Height, cm | 168.00 (162.00, 175.00) | 167.00 (162.00, 174.00) | 171.00 (165.00, 176.00) | **0.33 (0.26, 0.39)** |
| WC, cm | 81.00 (74.00, 88.00) | 79.00 (72.00, 86.00) | 87.00 (81.00, 93.00) | **0.89 (0.82, 0.96)** |
| Weight, kg | 65.00 (57.00, 74.00) | 63.00 (55.00, 71.00) | 74.00 (65.00, 81.00) | **0.87 (0.80, 0.93)** |
| BMI, kg/m² | 22.99 (21.07, 25.14) | 22.41 (20.57, 24.34) | 25.08 (23.15, 27.04) | **0.90 (0.83, 0.96)** |
| Smoking, n (%) | 814 (16) | 602 (15) | 212 (18) | **0.07 (0.00, 0.13)** |
| Alcohol consumption, n (%) | 752 (15) | 584 (15) | 168 (14) | **0.02 (0.04, 0.09)** |
| Medication use, n (%) | 393 (7.6) | 240 (6.0) | 153 (13) | **0.23 (0.17, 0.30)** |
| Type 2 diabetes, n (%) | 225 (4.3) | 147 (3.7) | 78 (6.5) | **0.13 (0.06, 0.19)** |
| Dyslipidemia, n (%) | 1,168 (23) | 716 (18) | 452 (38) | **0.45 (0.38, 0.51)** |
| Hypertension, n (%) | 791 (15) | 524 (13) | 267 (22) | **0.24 (0.17, 0.30)** |
| Neutrophils, ×10⁹/L | 3.26 (2.66, 4.00) | 3.19 (2.58, 3.93) | 3.48 (2.91, 4.20) | **0.29 (0.22, 0.35)** |
| Lymphocytes, ×10⁹/L | 1.86 (1.56, 2.22) | 1.84 (1.54, 2.18) | 1.97 (1.64, 2.36) | **0.26 (0.20, 0.33)** |
| Monocytes, ×10⁹/L | 0.32 (0.25, 0.40) | 0.31 (0.25, 0.39) | 0.34 (0.26, 0.42) | **0.20 (0.13, 0.26)** |
| WBC, ×10⁹/L | 5.64 (4.87, 6.62) | 5.53 (4.76, 6.46) | 6.00 (5.22, 7.03) | **0.35 (0.29, 0.42)** |
| RBC, ×10¹²/L | 4.73 (4.43, 5.07) | 4.67 (4.39, 5.01) | 4.91 (4.60, 5.23) | **0.47 (0.40, 0.53)** |
| Hemoglobin, g/L | 143.00 (133.00, 155.00) | 141.00 (131.00, 153.00) | 150.00 (138.00, 160.00) | **0.45 (0.39, 0.52)** |
| Platelets, ×10⁹/L | 237.00 (205.00, 274.00) | 236.00 (205.00, 273.00) | 237.00 (206.00, 277.50) | **0.06 (0.01, 0.12)** |
| ALT, U/L | 16.90 (12.91, 23.16) | 16.07 (12.28, 21.71) | 20.08 (15.32, 27.35) | **0.47 (0.41, 0.54)** |
| AST, U/L | 19.15 (16.46, 22.73) | 18.98 (16.32, 22.42) | 19.78 (16.90, 23.65) | **0.16 (0.10, 0.23)** |
| Uric acid, μmol/L | 331.00 (273.94, 398.19) | 316.37 (265.93, 381.65) | 375.77 (316.05, 436.02) | **0.58 (0.52, 0.65)** |
| Creatinine, μmol/L | 65.73 (56.09, 78.02) | 63.99 (55.54, 76.69) | 71.89 (59.58, 81.49) | **0.33 (0.26, 0.39)** |
| eGFR, mL/min/1.73m² | 109.85 (100.67, 117.64) | 110.33 (101.02, 118.29) | 108.28 (99.44, 115.30) | **0.16 (0.10, 0.22)** |
| Glucose, mmol/L | 5.40 (5.13, 5.73) | 5.36 (5.10, 5.67) | 5.53 (5.22, 5.89) | **0.34 (0.28, 0.41)** |
| Total cholesterol, mmol/L | 4.88 (4.31, 5.52) | 4.86 (4.30, 5.49) | 4.96 (4.34, 5.62) | **0.08 (0.02, 0.14)** |
| Triglycerides, mmol/L | 1.27 (0.93, 1.72) | 1.19 (0.88, 1.59) | 1.58 (1.19, 2.09) | **0.64 (0.57, 0.70)** |
| HDL-C, mmol/L | 1.39 (1.18, 1.63) | 1.45 (1.24, 1.67) | 1.21 (1.06, 1.43) | **0.68 (0.62, 0.75)** |
| LDL-C, mmol/L | 2.67 (2.20, 3.19) | 2.63 (2.17, 3.15) | 2.78 (2.31, 3.29) | **0.19 (0.12, 0.25)** |
| Homocysteine, μmol/L | 9.30 (7.40, 12.40) | 9.10 (7.30, 11.70) | 9.80 (7.90, 14.30) | **0.35 (0.29, 0.42)** |
| Hyperhomocysteinemia, n (%) | 728 (14) | 462 (12) | 266 (22) | **0.28 (0.22, 0.35)** |

**Notes:** SMD = Standardized Mean Difference; CI = Confidence Interval.

**Abbreviations:** SBP, systolic blood pressure; DBP, diastolic blood pressure; WC, waist circumference; BMI, body mass index; WBC, white blood cell; RBC, red blood cell; ALT, alanine aminotransferase; AST, aspartate aminotransferase; eGFR, estimated glomerular filtration rate; HDL-C, high-density lipoprotein cholesterol; LDL-C, low-density lipoprotein cholesterol; MASLD, Metabolic Dysfunction-Associated Steatotic Liver Disease.

**Supplementary Table 3. Ulticollinearity Test using Variance Inflation Factor**

| **Variable** | **VIF Value** |
| --- | --- |
| hcy | 1.409324 |
| age | 2.311700 |
| sex | 1.973206 |
| BMI | 1.266265 |
| smoking | 1.012284 |
| drinking | 1.009848 |
| ua | 1.878621 |
| drug | 1.695374 |
| T2DM | 1.190551 |
| hypertension | 1.638624 |
| dyslipidemia | 1.095281 |
| egfr | 2.262188 |

Abbreviations: Hcy, Homocysteine; SUA, Serum, Uric Acid; T2DM, Type 2 Diabetes Mellitus.

**Supplementary Table 4. Baseline biochemical characteristics according to homocysteine categories**

| **Characteristic** | **Overall**  **(N = 5,184)** | **Hcy <10 μmol/L**  **(N = 3,000)** | **Hcy 10–15 μmol/L**  **(N = 1,456)** | **Hcy >15 μmol/L**  **(N = 728)** | **SMD**  **(<10 vs 10–15)** | **SMD**  **(>15 vs 10–15)** | **SMD**  **(<10 vs >15)** |
| --- | --- | --- | --- | --- | --- | --- | --- |
| BMI, kg/m² | 22.99 (21.07, 25.14) | 22.57 (20.70, 24.65) | 23.51 (21.38, 25.45) | 23.92 (22.07, 25.98) | 0.225 | 0.145 | 0.377 |
| Triglycerides, mmol/L | 1.27 (0.93, 1.72) | 1.20 (0.89, 1.63) | 1.35 (0.97, 1.78) | 1.46 (1.07, 1.91) | 0.204 | 0.185 | 0.402 |
| HDL-C, mmol/L | 1.39 (1.18, 1.63) | 1.45 (1.25, 1.69) | 1.34 (1.13, 1.58) | 1.24 (1.07, 1.45) | 0.322 | 0.332 | 0.659 |
| LDL-C, mmol/L | 2.67 (2.20, 3.19) | 2.61 (2.14, 3.11) | 2.69 (2.26, 3.26) | 2.83 (2.32, 3.35) | 0.152 | 0.114 | 0.266 |
| ALT, U/L | 16.90 (12.91, 23.16) | 16.03 (12.26, 21.84) | 17.87 (13.49, 24.28) | 18.86 (14.42, 26.00) | 0.213 | 0.153 | 0.375 |
| AST, U/L | 19.15 (16.46, 22.73) | 18.68 (16.03, 22.16) | 19.93 (17.04, 23.42) | 19.64 (16.99, 23.61) | 0.219 | 0.003 | 0.222 |
| Glucose, mmol/L | 5.40 (5.13, 5.73) | 5.35 (5.10, 5.65) | 5.46 (5.18, 5.81) | 5.50 (5.18, 5.82) | 0.235 | 0.053 | 0.298 |
| Uric acid, μmol/L | 331.00 (273.94, 398.19) | 302.05 (258.17, 363.10) | 362.80 (304.93, 419.80) | 394.09 (336.40, 456.40) | 0.620 | 0.373 | 1.013 |
| Creatinine, μmol/L | 65.73 (56.09, 78.02) | 59.52 (53.31, 69.59) | 73.19 (62.89, 82.87) | 78.96 (70.80, 87.62) | 0.870 | 0.436 | 1.367 |
| eGFR, mL/min/1.73 m² | 109.85 (100.67, 117.64) | 112.74 (105.21, 119.39) | 105.54 (96.20, 113.70) | 104.49 (94.26, 113.94) | 0.614 | 0.103 | 0.731 |

**Abbreviations:** Hcy, homocysteine; BMI, body mass index; HDL-C, high-density lipoprotein cholesterol; LDL-C, low-density lipoprotein cholesterol; ALT, alanine aminotransferase; AST, aspartate aminotransferase; eGFR, estimated glomerular filtration rate. Data are presented as median (Q1, Q3). Standardized mean differences (SMDs) are shown for pairwise comparisons.

**Supplementary Table 5. Sensitivity Analyses for Homocysteine and MASLD Risk**

| **Variable**^1^ | **Excluding Drug Users** | | **Excluding Events <2 Years** | |
| --- | --- | --- | --- | --- |
|  | **HR (95% *CI*)** | ***p*-value** | **HR (95% *CI*)** | ***p*-value** |
| Per SD increment | 1.41 (1.12 to 1.78) | 0.003 | 1.16 (1.06 to 1.27) | 0.001 |
| Hyperhomocysteinemia | 1.57 (1.35 to 1.84) | <0.001 | 1.41 (1.12 to 1.78) | 0.003 |

Notes: Model Adjusted for age, sex, smoking, drinking, drug use, T2DM, hypertension, dyslipidemia, uric acid, BMI, and eGFR. HR = Hazard Ratio, CI = Confidence Interval

Abbreviations: T2DM, Type 2 Diabetes Mellitus; BMI, body mass index; MASLD, Metabolic Dysfunction-Associated Steatotic Liver Disease;
